# Domain Requirements and Sequence Specificity of DNA Binding for the Forkhead Transcription Factor FOXP3

**DOI:** 10.1371/journal.pone.0008109

**Published:** 2009-12-01

**Authors:** Kian Peng Koh, Mark S. Sundrud, Anjana Rao

**Affiliations:** Department of Pathology, Harvard Medical School and Immune Disease Institute, Boston, Massachusetts, United States of America; New York University School of Medicine, United States of America

## Abstract

The forkhead, winged-helix transcription factor FOXP3 is preferentially expressed in T regulatory (Treg) cells and is critical for their immunosuppressive function. Mutations that abolish FOXP3 function lead to systemic autoimmunity in mice and humans. However, the manner by which FOXP3 recognizes cognate DNA elements is unclear. Here we identify an *in vitro* optimized DNA sequence to assess FOXP3 DNA binding by electrophoretic mobility shift assay (EMSA). The optimized sequence contains two tandem copies of a core DNA element resembling, but not identical to, the canonical forkhead (FKH) binding element. The tandem nature of this optimized FOXP3-binding oligonucleotide suggests a requirement for multimerization, and EMSA experiments confirm that both the DNA-binding FKH domain and an intact leucine-zipper domain, which mediates homo-multimerization of FOXP3, are required for DNA binding. These results establish a practical framework for understanding the molecular basis by which FOXP3 regulates gene transcription and programs Treg suppressive function.

## Introduction

Forkhead box (FOX) transcription factors are a large and functionally diverse family of transcription factors, with over 100 members in mammals (reviewed in [1]). Named after the *forkhead* gene product in *Drosophila melanogaster*, the founding members of the mammalian FOX family belong to the hepatic nuclear factor-3 (HNF3/FOXA) family, which regulate the development of metabolic tissues such as the pancreas and liver [2], [3]. Many FOX transcription factors are tissue-specific regulators of development (reviewed in [4]): hair formation and keratinocyte differentiation are regulated by Foxn1 [5], cell growth and insulin responsiveness by Foxo1 [6], craniopharyngeal development by FOXE1 [4], speech and language patterning by FOXP2 [7], and auditory function by Foxi1 [8]. Additionally, several FOX proteins play key roles in the development, homeostasis and function of immune cells (reviewed in [9]). Foxo1, Foxo3 and Foxp1 all regulate B cell ontogeny, possibly through direct transcriptional regulation of the *Rag1/2* locus [10]–[13]. In T cells, genetic ablation of either Foxj1 or Foxo3 precipitates a lymphoproliferative phenotype associated with variable autoimmune pathology [14], [15], suggesting that these FOX proteins negatively regulate T cell activation.

Foxp3 (denoted FOXP3 in humans) displays one of the more striking functions of a FOX protein within the immune system. FOXP3 is selectively expressed by a subset of CD4+ T cells, known as T regulatory (Treg) cells, which suppress effector T cell function in response to self or foreign antigens (reviewed in [9], [16], [17]. FOXP3 is encoded on the X-chromosome, thus loss or mutation of FOXP3 is not deleterious in females. However, mutations within the *FOXP3* gene in male infants are causally linked to IPEX (immune dysregulation, polyendocrinopathy, enteropathy, X-linked), a severe perinatal autoimmune syndrome resulting from defects in Treg development and consequent activation of conventional T cells with specificity for self-antigens [18], [19]. IPEX patients develop lymphadenopathy, splenomegaly, hyper-IgE production, variable hyperglycemia and lymphocytic infiltrates into the lung, skin, pancreas and liver [18]–[20]. The autoimmune phenotype of IPEX patients is phenocopied in male *scurfy* (Foxp3*^sf^*) mice, which harbor a spontaneous mutation in the *Foxp3* gene [16], [21], [22]. Male mice in which *Foxp3* is conditionally deleted in T cells using Cre recombinase expressed under the control of the CD4 promoter (CD4-Cre) develop a similar severe autoimmune phenotype [23], as do adult mice in which Foxp3-expressing Treg cells are acutely ablated [24]. These observations have resulted in much focus on the transcriptional regulatory function of FOXP3.

FOXP3 contains a large (∼181 aa) amino-terminal region required for transcriptional activation and repression, a central C2H2 zinc-finger domain to which no specific function has yet been ascribed, a leucine-zipper domain implicated in multimer formation and suppressor function, and a C-terminal forkhead (FKH) domain that mediates DNA-binding by FOX proteins [9], [16]. FOXP3 can associate with auxiliary transcription factors such as NFAT, AML1/Runx1, IRF4 (not shown to physically interact with Foxp3) and NF-κB to drive the transcription of specific subsets of FOXP3 target genes [25]–[28]. These associations have been largely observed via co-immunoprecipitation and chromatin immunoprecipitation (ChIP) assays. ChIP assays can localize transcription factor binding to relatively large (200–500 bp) regions of DNA, and have proved useful in confirming or revealing target promoters likely to be directly regulated by FOXP3 and its transcriptional partners. Large-scale ‘ChIP-chip’ assays, in which DNA occupied by specific transcription factors is immunoprecipitated and hybridized to genome-wide tiling arrays, have been used to identify DNA elements likely to bind FOXP3 *in vivo*, either alone or in complex with transcriptional partners [29], [30]. However, these analyses have yet to be confirmed by *in vitro* assays that directly assess FOXP3:DNA-binding.

Using a systematic series of electrophoretic mobility shift assays (EMSA), we have explored the basis for the sequence-specific DNA-binding by FOXP3. We show that a fragment lacking the first 181 amino acids of FOXP3 (Foxp3-ΔN) binds DNA far more robustly than full-length FOXP3. Efficient DNA binding by this fragment requires both the leucine zipper and FKH domains. The preferred oligonucleotide defined by EMSA assays as a high-affinity FOXP3-binding site contains two tandem FOXP elements, which are similar to, but somewhat divergent from, the classic forkhead-binding sites previously identified for HNF3/FOXA proteins. Based on the tandem nature of optimal FOXP3 binding sequences, together with the requirement for the leucine-zipper motif for DNA binding, we propose that FOXP3 binds DNA with high affinity as at least a dimer and that the N-terminal region has an autoinhibitory effect. Collectively, these results lay the foundation for understanding how FOXP3 controls the immunosuppressive transcriptional program of Treg cells.

## Methods

### Plasmids

cDNAs encoding full length mouse Foxp1A (generous gift of Dr. Phil Tucker, University of Texas-Austin) or full length human FOXP3, as well as truncated and/or mutated versions were cloned into the expression vector pcDNA3.1(+). Point mutations/deletions were generated in the constructs using the Quickchange site-directed mutagenesis system (Strategene).

### Electrophoretic Mobility Shift Assays

The following oligonucleotide sequences were used as probes in gel-shift assays (one strand shown with putative binding sites underlined):

A: 5′-CAAGGTAAACAAGACAACACAAATA A-3′;

A’(A1-A1): 5′-CAAGGTAAACAAGACA ACGTAAACAA-3′ ;

A″ (A2-A2): 5′-CAAGACA AATAAGACAACACAAATAA-3′;

A’(AT): 5′-CAAGATAAACAAGACAACATAAACAA-3′;

A’(GC): 5′-CAAGGCAAACAAGACAACGCAA ACAA-3′;

A’(AC): 5′-CAAGACAAACAAGAC AACACAAACAA-3′;

A’ (29 bp): 5′-CAAGGTA AACAAGACAACGTAAACAAGTC-3′;

A’ (25 bp): 5′-CAAGGTAAACAAGAGTAAACAAGTC-3′;

A’ (35 bp): 5′-CAAGGTAAACAAGACAACACG ATTGTAAACAAGTC-3′.

Single-stranded oligonucleotides containing the consensus Foxp1/FOXP3 binding sites were annealed with their complementary strands and purified on 12% polyacrylamide gels for use as probes in electrophoretic mobility-shift assays (EMSA). Probes were end-labeled with γ^32^P-ATP using T4 polynucleotide kinase in accordance with manufacturers' instructions. *In vitro*-translated proteins were generated using the TNT reticulocyte lysate system (Promega). Binding reactions were performed at room temperature for 20 minutes using 5 µl of *in vitro*-translated proteins and approximately 10,000–20,000 c.p.m. (∼0.1–0.5 ng) of ^32^P-end labeled probes in 20 µl. The final concentration of components of the binding buffer for all EMSA experiments were: 12 mM HEPES pH 7.5, 100 mM NaCl, 1 mM DTT, 1 mM EDTA, 12% glycerol and 20 µg/ml poly(dI)-poly(dC). DNA-protein complexes were separated from free probe by electrophoresis in a 5% polyacrylamide, TBE gel containing 1% glycerol. Dried gels were exposed to autoradiography film between 1 hour to overnight at room temperature. Quantification of band intensities were performed on autoradiograms from 1 hour exposures using the software ImageJ.

### Western Blot

Equal quantities of *in vitro*-translated protein lysates were resolved by SDS-polyacrylamide gel electrophoresis and transferred to nitrocellulose membranes (Whatman). Immunoblots were performed using either a monoclonal antibody against HA (for HA-tagged Foxp1), a monoclonal antibody 1G1 raised against the FKH domain of Foxp1 (generously provided by Dr. Philip Tucker) or a polyclonal rabbit antisera raised against full-length human FOXP3 (generously provided by Dr. Steven Ziegler). Antibodies were diluted in Tris-buffered saline containing 0.1% Tween-20 and 3% non-fat dry milk. Secondary horseradish peroxidase-conjugated goat anti-mouse or anti-rabbit secondary antibodies (Sigma-Aldrich) were used to detect primary antibody binding, followed by detection with an enhanced chemiluminescence (ECL) reagent (Perkin-Elmer).

## Results

All FOX transcription factors share a common winged-helix DNA-binding domain of approximately 100 amino acids known as the forkhead (FKH) domain [1], [4], [9]. HNF3/FOXA proteins bind as monomers to DNA elements with the consensus sequence 5′–ATAACT–3′ [32], [33]; however, primary sequence analyses of their FKH domains, and hence their putative sequence specificity for DNA, show a significant degree of divergence from the FKH domains of other FOX proteins [1]. Indeed Foxp1A, a close relative of FOXP3, was found to prefer modified FKH/FOX DNA elements (5′ TATTTg/aTg/aTT-3′) or its complement, 5′–AAc/tAc/tAAATA-3′) in a PCR-based site-selection assay from which the “A” oligonucleotide containing the preferred Foxp1 binding site was derived [34].

**Figure 1:**
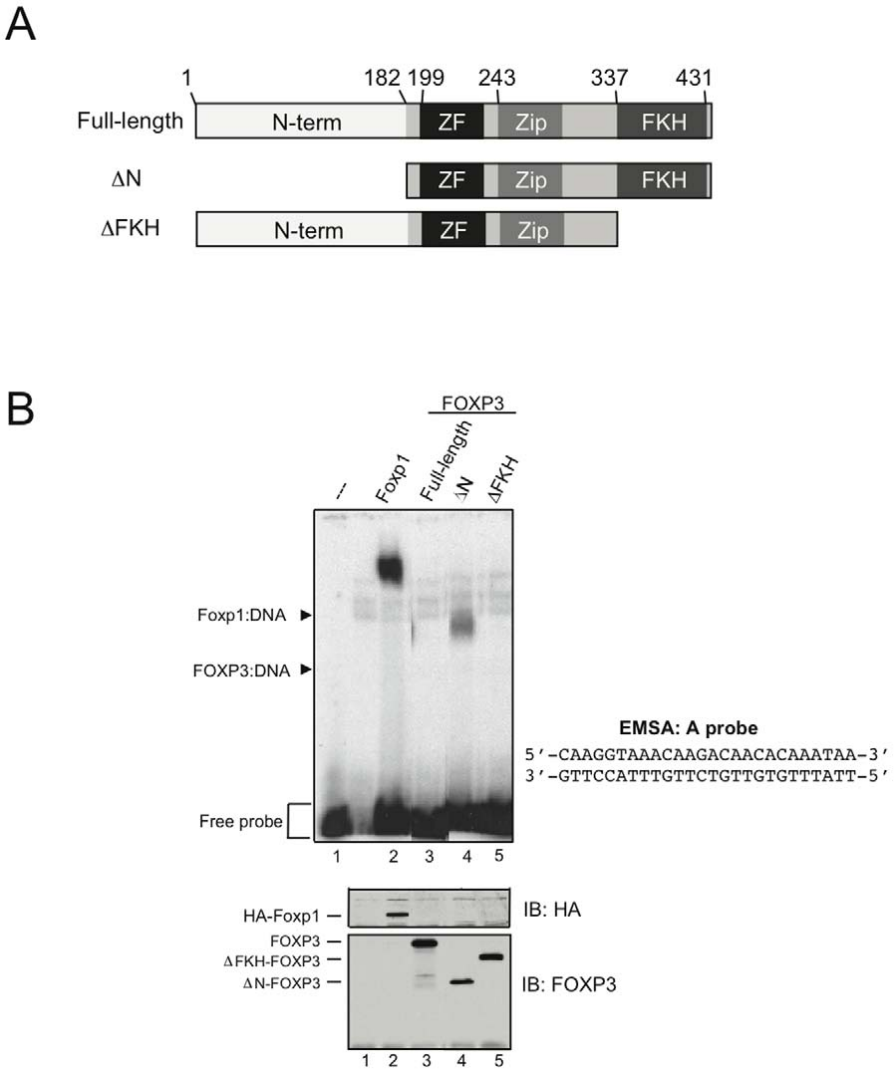
Deletion of the FOXP3 N-terminal region allows binding to a Foxp1-like DNA element. (A) Fragments of human FOXP3 translated *in vitro* (see materials) for use in EMSA assays. Residue numbering is listed above full-length FOXP3. N-term – the N-terminal region (1–181) of FOXP3, ZF – zinc finger, Zip – leucine zipper, FKH – forkhead domain. (B) *Top* – Full-length Foxp1 or FOXP3 fragments were expressed and incubated with radiolabelled A probe. Arrowheads indicate Protein:DNA complexes and free probe. *Bottom* – Anti-HA monoclonal antibodies or FOXP3 antiserum were used to detect expression of Foxp1 or FOXP3 fragments, respectively, by western blotting. Arrowheads indicate the position of each construct. Data are representative of at least 2 independent experiments.

We previously showed using a nonradioactive EMSA format – in which DNA and protein reactants are present at micromolar rather than nanomolar concentrations – that recombinant FOXP3-FKH domain expressed in bacteria bound very weakly on its own to the ARRE2 sequence from the mouse IL-2 promoter but formed a cooperative complex with recombinant NFAT1 DNA-binding domain on DNA [25]. Binding of the isolated FKH domain of FOXP3 to the ARRE2 sequence or the A oligonucleotide (see below) could not be detected in radioactive EMSA assays (data not shown), suggesting that other regions in FOXP3 are required for optimal DNA binding. To explore this possibility, we synthesized murine full-length HA-tagged Foxp1A (referred to throughout as Foxp1), human full-length FOXP3, or defined fragments of FOXP3 (Figure 1A), by *in vitro*-transcription/translation in reticulocyte lysates. To compare the ability of these proteins to bind DNA *in vitro*, we used the A oligonucleotide (sequence shown in Figure 1B), which contains the Foxp1 consensus sequence [34], as the starting probe in radioactive EMSA. All proteins were robustly expressed (Figure 1B, bottom). As expected, full-length Foxp1 bound strongly to the A probe (Figure 1B, lane 2), but surprisingly, full-length FOXP3 did not (lane 3). In contrast, a fragment lacking the first 181 N-terminal amino acids of FOXP3, here designated FOXP3-ΔN [25], bound to the A probe, although more weakly than that observed for Foxp1 (Figure 1B, lane 4). Binding to the A probe was specific, as neither Foxp1 nor FOXP3-ΔN bound to a labeled oligonucleotide from the variable 1 region of the immunoglobulin promoter (V1P), which contains a canonical FOX consensus sequence defined for the HNF3/FOXA proteins (data not shown) [33]. FOXP3-ΔN was thus used in subsequent experiments to define the DNA-binding specificity of FOXP3.

**Figure 2:**
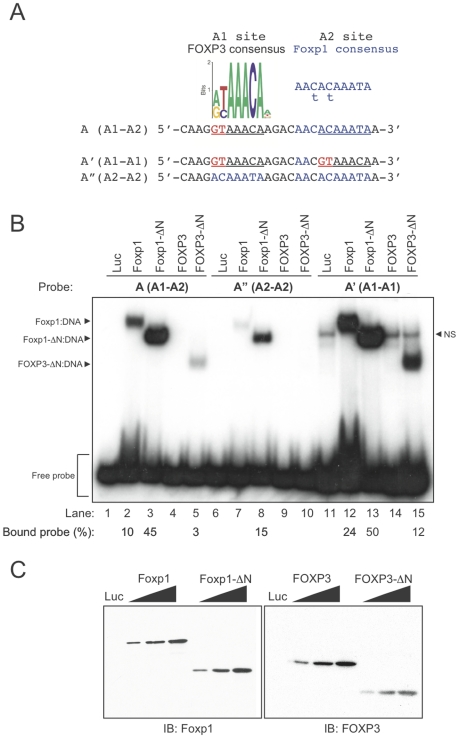
DNA binding specificities of Foxp1 and FOXP3. (A) Sequences of the A probe [34], A′ and A″ oligonucleotides used for EMSA experiments. The 5′ putative FOXP3-binding site (A1) is similar to a predicted Foxp3 binding site [29], [30] (see insert adapted from [30] below sequence text). The 3′ binding site (A2) represents the Foxp1 consensus site (blue text) as determined previously [34]. A′ has two putative FOXP3-binding sites (A1-A1). A″ has two putative Foxp1 sites (A2-A2). (B) *In vitro*-translated firefly luciferase (Luc), Foxp1 (full-length or ΔN) or FOXP3 (full-length or ΔN) were incubated with each labeled probe as indicated. Protein:DNA complexes and free probe are indicated at left (by arrowhead in one case and a square bracket in the other). NS – non-specific. Quantification values of bound probe (shown as % bound of total detected probe in each lane) were indicated below the lanes. (D) Expression of Foxp1 or FOXP3 constructs was evaluated by western blotting using anti-Foxp1 monoclonal or anti-FOXP3 polyclonal antisera respectively. Loading amounts of *in vitro*-translated lysates were 4 µl for Luc and 1 µl, 2 µl and 4 µl for the Foxp1 and FOXP3 constructs as in (C). Data are representative of at least two independent experiments.

The weak binding of FOXP3-ΔN to the A probe prompted us to derive an optimized sequence for FOXP3 DNA binding. Sequence inspection of the A probe revealed two potential FOXP-binding elements separated by a 7-nt spacer (Figure 2A). The 5′ element (5′–GTAAACA-3′, here designated A1) matched a computationally-identified FOXP3 binding element obtained via ChIP-chip experiments (G/A T/c AAACA, Figure 2A) [*30*]. The 3′ binding site (5′–AACACAAATA, here designated A2) was previously defined as the Foxp1 consensus site (5′-AA C/t A C/t AAATA, Figure 2A) [34]. FOXP3-ΔN contains a leucine-zipper domain reported to mediate homotypic interactions, that is mutated in a subset of IPEX patients [9], [16], suggesting that the A1 and A2 sequences might interact independently with FOXP3 forkhead domains within a FOXP3 multimer. To test this hypothesis and determine whether FOXP proteins discriminated between these sites, we synthesized two new double-stranded oligonucleotides, A′ or A″, containing two A1 or A2 elements, respectively (Figure 2A), and assessed their binding to *in vitro*-translated Foxp1, Foxp1-ΔN, FOXP3, or FOXP3-ΔN proteins in radioactive EMSA assays. For both Foxp1 and FOXP3, the ΔN versions bound DNA more effectively than the full-length proteins (Figure 2B), even though they were not over-expressed relative to the full-length proteins (Figure 2C). In fact, DNA binding by full-length FOXP3 was not detectable with any of the three probes (Figure 2B). Both Foxp1-ΔN and FOXP3-ΔN displayed diminished binding to the A″ (A2-A2) probe but enhanced binding to the A′ (A1-A1) probe, giving an order of preference for both proteins of A′ (A1-A1) > A (A1-A2) > A″ (A2-A2) (Figure 2B).

The lack of detectable binding of FOXP3-ΔN to the A″ (A2-A2) probe, despite binding to A′ (A1-A1) and A (A1-A2), suggests that the A1 sequence (GTAAACA) is essential for FOXP3 DNA binding. The diminished binding of full-length Foxp1 and Foxp1-ΔN to A″ (A2-A2) was surprising, given that the probe contains the defined Foxp1 consensus element [34] duplicated in tandem. These results suggest that the strong binding of Foxp1 to the A oligonucleotide was in fact facilitated by the presence of the A1 element (GTAAACA) serving as a stronger Foxp1 consensus site than A2 (ACAAATA).

**Figure 3:**
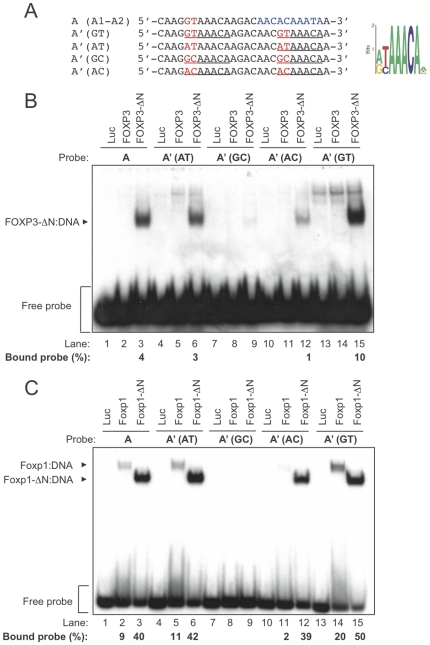
Definition of the FOXP3 consensus binding site. (A) *Left* – Using the A′ oligonucleotide sequence as a reference, the two 5′ nucleotides were randomized within each of the two putative FOXP3 binding sites. Each FOXP3 binding site is underlined and the randomized 5′ dinucleotide motifs are indicated by red text. *Right* – Computationally-determined putative FOXP3 binding site as in the figure 2 legend. (B) *In vitro*-translated firefly luciferase (Luc), full-length FOXP3 or FOXP3-ΔN were incubated with each labeled probe as indicated. Protein:DNA complexes and free probe are indicated by arrowhead and square brackets as in the legend to Figure 2. (C) The same experiment as described above in Figure 3B was performed with *in vitro*-translated full-length Foxp1 or Foxp1-ΔN. Quantification values of bound probe were indicated below the lanes. These data represent at least 2 independent experiments.

Since the A1 sequence (GTAAACA) is only one of four possible sequences derived from the computationally-identified FOXP3 consensus site (G/A T/c AAACA) [*30*], we repeated the EMSA assays using A′ (A1-A1) oligonucleotide probes that contained all the possible combinations of these preferred nucleotides: AT, AC, GT and GC (Figure 3A). FOXP3-ΔN showed a strong preference for duplicated GTAAACA sequences, with binding affinity more than doubled compared to the original A probe containing only one copy of GTAAACA, or the oligonucleotide containing two ATAAACA sequences (Figure 3B). FOXP3-ΔN binding to ACAAACA sequences was further decreased, and binding was altogether abolished to GCAAACA sequences (Figure 3B). These data suggest that FOXP3-ΔN:DNA-binding is tightly regulated by the two 5′ nucleotides within its binding sites and gives an order of preference of GT>AT>AC (Figure 3B). Foxp1 displayed similar preferences for its DNA-binding sites (Fig. 3C). In this case, however, the differential preference for GT, AT and AC was less pronounced, whereas binding to sequence starting with GC was again very weak. Collectively, these experiments define the core consensus binding element for both FOXP proteins as two tandem copies of the sequence 5′–GTAAACA–3′.

**Figure 4:**
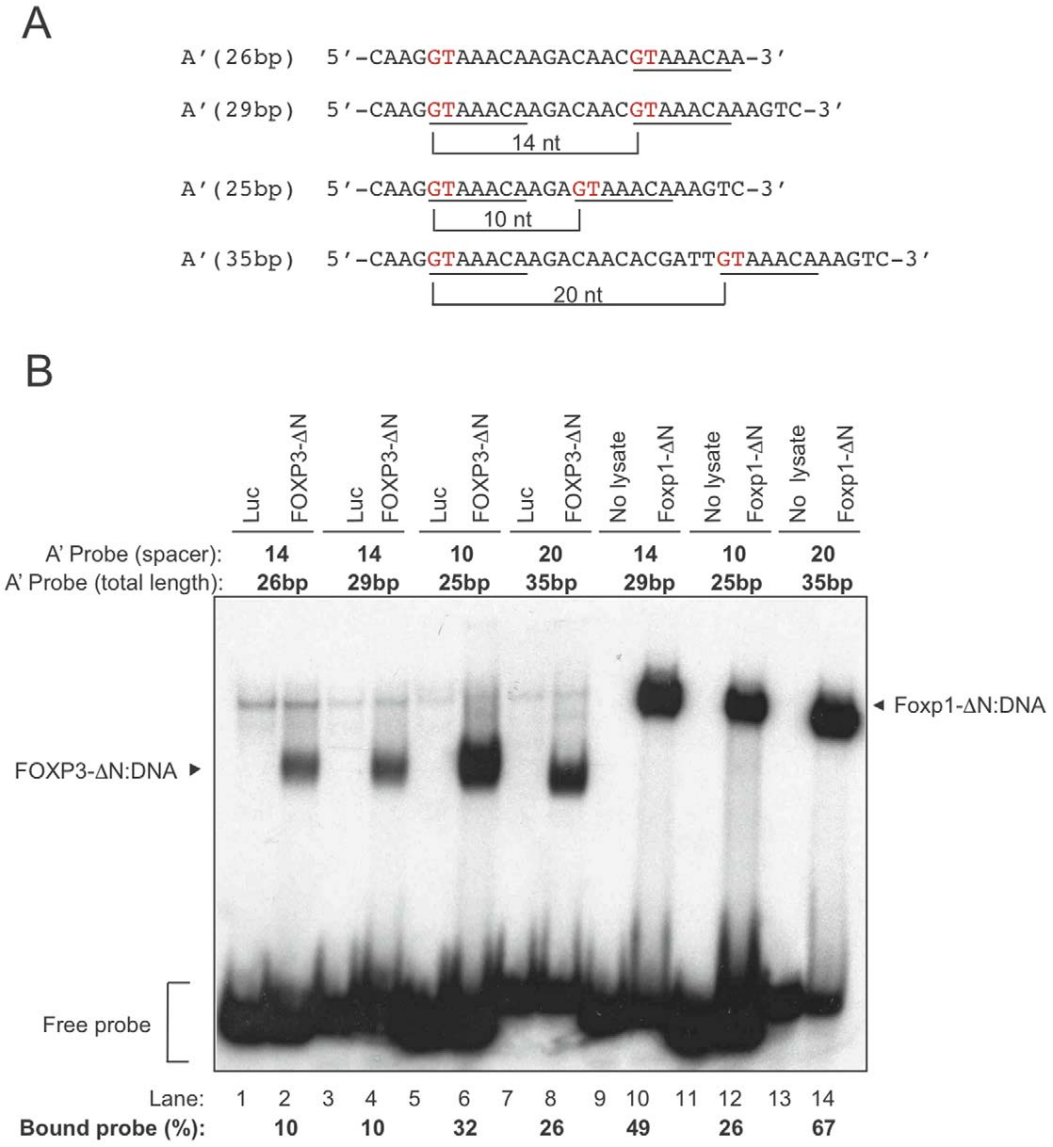
FOXP3:DNA binding is regulated by spacing between tandem elements. (A) Sequences of the oligonucleotides used as probes in EMSA assays. Each FOXP3 binding site is underlined with the first two nucleotides of each site highlighted in red text. The space (in number of nucleotides) separating the 5′ ends of the two binding sites is indicated below each sequence. The total number of base pairs in each sequence is listed next to the probe name in parentheses. (B) *In vitro*-translated firefly luciferase (Luc), or FOXP3-ΔN were incubated with each labeled probe as indicated. Protein:DNA complexes and free probe are indicated. Quantification values of bound probe were indicated below the lanes. All data shown are representative of at least 2 separate experiments.

We next asked whether the spacing between the two core binding sites was important for FOXP3:DNA binding. For this we used synthetic oligonucleotides in which the 5′ ends of the two FOXP3 consensus elements were separated by 14 base-pairs (bp), as in the original A probe, or alternatively by 10 or 20 bp, corresponding to one or two complete turns of the DNA helix respectively, which would place the two sites on the same side of the DNA (Figure 4A). We found that shortening the spacer length between the two binding sites to 10 nucleotides, or lengthening the spacing to 20 nucleotides, increased FOXP3-ΔN DNA binding by ∼3-fold (Figure 4B). In contrast, Foxp1-ΔN preferred the longer spacing, with the 20-bp spacing somewhat preferred over the 14-bp spacing originally selected by PCR-based site selection approaches (Figure 4B), but showed lower binding when the core sites were spaced by 10 bp. These data suggest that the optimal FOXP3 binding element contains two 5′–GTAAACA–3′ sites that are presented on the same side of a DNA helix and that can be as close as 10 bp apart. However, the structural requirements for binding differ from those of Foxp1, which seems to prefer binding sites spaced apart by more than a single helical turn.

**Figure 5:**
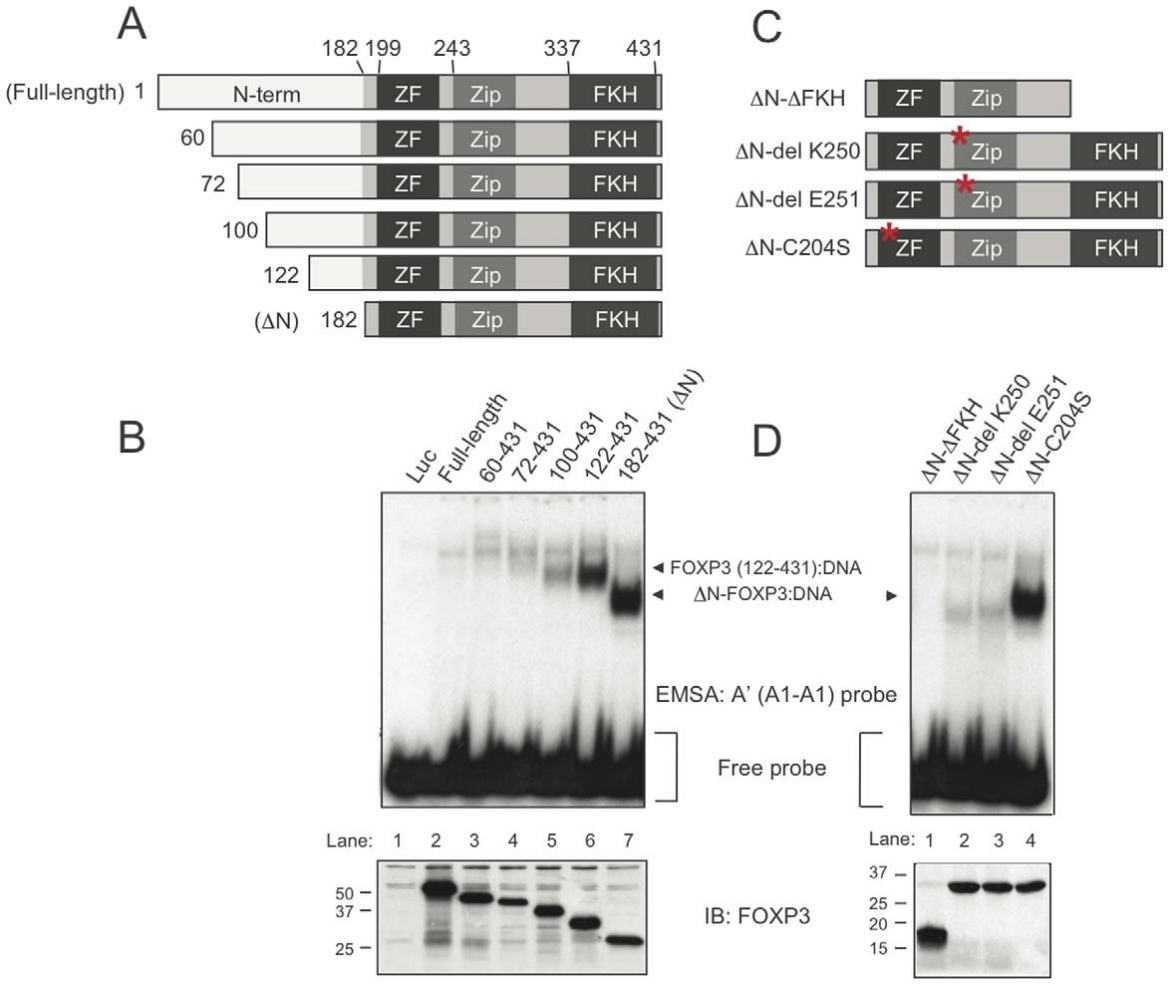
DNA binding by N-terminally truncated or point-mutated FOXP3. (A) Schematic representation of N-terminally truncated FOXP3 proteins used in EMSA assays. The position of the starting residue is listed next to each construct. (B) *Top* – Firefly luciferase (Luc), full-length FOXP3, or FOXP3 N-terminal truncation mutants were *in vitro*-translated and incubated with labeled A′ (A1-A1) probe. Arrowheads indicate Protein:DNA complexes. *Bottom* – expression of full-length FOXP3 or N-terminal FOXP3 mutants was determined by western blotting as in the legend to Figure 1. (C) Diagram of the FOXP3-ΔN fragments used to determine the requirement of each domain for DNA binding. A single amino acid substitution within the zinc-finger domain (C204S), or single amino acid deletions within the leucine-zipper domain (del K250, del E251) are indicated by red asterisks. (D) *Top* – *In vitro*-translated firefly luciferase (Luc), or FOXP3-ΔN mutants were incubated with labeled A′ (A1-A1) probe. Arrowheads indicate Protein:DNA complexes and free probe. *Bottom* – expression of FOXP3-ΔN mutant proteins was determined by western blotting. The same results were obtained using the A′ (A1-A1) probe with 10-bp spacing described in Fig. 4. These data represent at least 2 independent experiments.

The fact that full-length FOXP3 did not bind even to the optimized A′ (A1-A1) probe suggested that the N-terminal region of FOXP3 has an autoinhibitory function that restricts DNA binding *in vitro*. To define the boundaries of this putative region, we *in vitro*-translated a series of FOXP3 proteins that all retained the zinc-finger, leucine-zipper and FKH domains, but in which the N-terminus was truncated to varying extents (Figure 5A, 5B-bottom). No binding could be detected even to the spacing-optimized A′(A1-A1) probe until the N-terminal 121 amino acids of FOXP3 were deleted. Truncation of the entire proline-rich N-terminus region until amino acid 181, giving rise to FOXP3-ΔN, was required for strongest binding to the probe (Figure 5B, lanes 6, 7).

In addition to the DNA-binding FKH domain, FOXP3-ΔN contains a zinc-finger of unknown function and a leucine-zipper domain reportedly involved in homo-multimerization (Figure 5C) [9], [16], [35]. As expected, deletion of the FKH domain eliminated DNA-binding by FOXP3-ΔN (Figure 5D, lane 1). Additionally, FOXP3-ΔN:DNA binding was abrogated by two IPEX mutations that affect the leucine-zipper domain (del K250 and del E251) [16], [35], [36] (Figure 5D, lanes 2, 3). These single amino acid deletions have been shown to abrogate FOXP3 multimerization [35], presumably by affecting the positioning of leucine residues along the face of the α-helical leucine-zipper domain. In contrast, DNA binding of FOXP3-ΔN was not influenced by substitution of a zinc-coordinating residue within the zinc-finger domain (C204S) (Figure 5D, lane 4); nor was binding of FOXP3-ΔN enhanced by including ZnSO_4_ in the binding reaction (data not shown). These data indicate that the FKH domain and an intact leucine-zipper are both required for optimal DNA-binding by FOXP3-ΔN, whereas the zinc-finger domain is dispensable.

## Discussion

FOXP3 prevents spontaneous autoimmunity by conferring on Treg cells the transcriptional profile responsible for their immune-suppressive activity. In this study we used a systematic series of EMSA assays to define the parameters that regulate high-affinity interactions between FOXP3 and cognate DNA elements. We show that the core FOXP3 consensus element contains the sequence 5′–(G/a)TAAACA–3′; this sequence is also preferred by the closely related transcription factor Foxp1, but diverges from the classical FKH/FOX consensus site (5′–ATAACT–3′) defined for the HNF3/FOXA proteins [32]–[34]. The consensus Foxp1/FOXP3 binding element we have defined here is substantiated by previous studies that identified Foxp3 binding sites throughout the genome via ChIP-chip [29], [30]. In these reports, the predicted Foxp3 binding sequence was 5′–(A/G)(T/C)AAACA–3′. Our analysis has further defined the sequence specificity of FOXP3 as strongly preferring thymine at position 2 and favoring guanine at position 1, although adenine is also tolerated.

The degenerate nature of FOXP3 binding sites *in vivo* [29], [30] may reflect the contributions of additional co-factors at specific loci. This hypothesis is supported by previous work showing that FOXP2 and FOXP3 can bind DNA at a non-consensus site in the *Il2* promoter (5′-TGTTTCA-3′) [25]. The complement of this sequence, 5′–TGAAACA–3′, matches the FOXP3 binding sequence defined here except for inversion of the order of the first two nucleotides [25], which on its own would be predicted to be non-permissive for FOXP3:DNA binding. However, this site is located immediately adjacent to an NFAT site in the composite ARRE-2 element that also binds NFAT:AP-1 complexes, and the strong cooperative complexes formed between NFAT:AP-1 and NFAT:FOXP3 at this composite element promote and inhibit *Il2* gene transcription respectively [25]. Thus, although FOXP3 binding *in vitro* is restricted to its defined consensus site and requires tandem binding elements, cooperative DNA binding by FOXP3 in complex with other transcription factors may stabilize FOXP3 binding at non-consensus sites [25], [26], [28].

Unlike the HNF3/FOXA proteins, which bind with high affinity as monomers to single consensus sites [33], we show here that Foxp1 and FOXP3 preferentially bind oligonucleotides containing two consensus sites arrayed in tandem. These results suggest that FOXP proteins bind DNA as at least a dimer [34]. In further support of this hypothesis, we find that DNA binding by FOXP3 requires the FKH domain together with an intact leucine-zipper domain, whereas the zinc-finger domain is dispensable. The leucine-zipper domain of FOXP3 mediates homotypic interactions [35], whereas HNF3/FOXA proteins lack a leucine-zipper [4], [9], [33]. Therefore, the presence of a leucine-zipper domain within FOX proteins correlates with their distinctive preference for tandem sequences in DNA. Indeed, at least two independent single amino acid deletions within the leucine-zipper of FOXP3 (del K250, del E251), both associated with IPEX [16], [36], fail to bind our optimized DNA sequence in EMSA assays (Figure 5D). Our data therefore suggest that the primary loss of function in these IPEX mutants relates to dimerization and DNA binding.

Interestingly, FOXP3:DNA binding *in vitro* was only detected upon removal of the N-terminal region (FOXP3-ΔN); truncating the corresponding N-terminal region of Foxp1 also enhanced DNA binding. A trivial possibility is that the N-terminal region is unstructured and interferes, in the *in vitro*-translated protein, with DNA binding or multimerization. Another, more interesting possibility is that the N-terminal region of FOXP3 possesses an autoinhibitory function, possibly regulating FOXP3:DNA binding indirectly. Sequence comparisons between the N-terminal regions of FOXP proteins indicate significant divergence (reviewed in [1]). For example, the Foxp1 N-terminal region contains a poly-glutamine (poly-Q) sequence that is absent from the N-terminus of FOXP3 [34]. Furthermore, this N-terminal region of FOXP3 is responsible for activation as well as repression of target genes [25], [35], and has been shown to interact with a number of auxiliary transcription factors and chromatin-modifying proteins [39]. Thus, N-terminal sequence divergence among FOXP proteins may serve to recruit unique protein complexes to target promoters, which in turn would dictate whether gene transcription is activated or repressed. Consistent with this notion, previous reports have shown that FOXP3:DNA binding is increased, in a cyclosporine A-sensitive manner, upon stimulation of T cells through the T cell antigen receptor [25], [30], [40]. A plausible hypothesis is that the N- terminal region of FOXP3 regulates DNA binding and transcriptional activity, either through co-factors that bind this region or through post-translational modification.

In summary, we have defined an optimal set of *in vitro* conditions to study FOXP3:DNA binding: (1) by removing the proline-rich N-terminal region of FOXP3, and (2) by using an optimized probe containing two consensus sites, 5′- GTAAACA-3′ separated by one or two turns of the DNA helix. Our findings will facilitate further structural studies of FOXP3 in complex with DNA, promoting a precise biochemical understanding of how FOXP3 binds to DNA, either alone or in cooperation with its transcriptional partners, to regulate the expression of target genes in Treg cells.
