# Correction: Domain Requirements and Sequence Specificity of DNA Binding for the Forkhead Transcription Factor FOXP3

**DOI:** 10.1371/annotation/651b136b-79cf-44d4-84c7-a35b1930d1ee

**Published:** 2010-01-23

**Authors:** Kian Peng Koh, Mark S. Sundrud, Anjana Rao

The following information was missing from the Funding section: KPK was supported by a postdoctoral fellowship from the American Heart Association. 

